# Chemosensory input from mouthparts in response to sexually dimorphic cuticular wax mediates male sexual discrimination in *Galerucella grisescens* (Coleoptera: Chrysomelidae)

**DOI:** 10.1038/s41598-023-49272-1

**Published:** 2023-12-08

**Authors:** Yuki Chiba, Shun Yosano, Masatoshi Hori

**Affiliations:** 1 Graduate School of Agricultural Science, Tohoku University, Sendai, Miyagi 980-8572 Japan; 2 Institute for Plant Protection, National Agriculture and Food Research Organization, Tsukuba, Ibaraki 305-8666 Japan

**Keywords:** Animal behaviour, Entomology

## Abstract

The surface of the insect body is covered with a hydrophobic layer called cuticular wax (CW). In addition to functioning as an anti-desiccation agent, CW is critical for chemical communication. It has been reported that in Chrysomelidae, males discriminate between sexes based on the sex-specific CW. However, little is known regarding the underlying sensory basis. Herein, we demonstrate that chemosensory input from mouthparts mediates sexual discrimination in male *Galerucella grisescens* (Chrysomelidae). Observations of mating behaviour, bioassays for CW, and chemical analyses revealed that *G. grisescens* possess qualitatively sexually dimorphic CW, and such compositional differences allow males to distinguish between sexes. Using electron microscopy, blocking male chemosensory organs, and electrophysiological experiments, we showed that male mouthparts bear chemosensory sensilla tuned to female CW components, and sensory input from them induces male aedeagal insertion, a common male behavioural response to females. Thus, detecting CW via mouthparts is essential for males to distinguish between sexes, consistent with the fact that males inspect conspecific individuals by licking their body surfaces. To our best knowledge, this is the first report describing the detailed functional roles of mouthparts in sexual discrimination in Coleoptera. We believe that this study will promote further studies on insect chemical communication.

## Introduction

Chemical cues are among the most widespread mating signals in insects, and many species rely on pheromones for intra-species communication. Insect pheromones can be roughly divided into two categories on the basis of their chemical properties: volatile pheromones, which contribute to long-range attraction; and non- or low-volatile pheromones, which are associated with close-range communication^1^. Close-range communication makes use of a hydrophobic layer that covers the body of insects, called cuticular wax (CW)^2^.

CW is composed of hardly volatile chemicals such as long-chain alcohols, ketones, esters, aldehydes, fatty acids, and hydrocarbons^3^. In addition to its role as a barrier against desiccation, the CW is also essential for chemical communication among various insect taxa^4^. Social insects such as ants, for example, possess caste- and colony-specific CW profiles, which contribute to their task-specialisation within the colony and nestmate discrimination^5^. CW is also important for chemical communication in solitary insects. Although several other functional roles have also been reported for the CW (e.g., host recognition cues in parasitic wasps^6^ and chemical mimicry in myrmecophilous insects^7,8^), its most common role is as a sex pheromone. In several insect orders, including Coleoptera^9–19^, Hymenoptera^20^, Diptera^21–23^, Orthoptera^24^, and Lepidoptera^25,26^, CW profiles are often sexually dimorphic, and it is believed that males can recognise whether the individuals they encounter are proper mates by detecting their CW.

In Chrysomelidae, both males and females are attracted to volatile male-produced aggregation pheromones^27–33^. However, at short distances, the CW is used for chemical communication. Previous studies have reported that female CW elicits male mating behaviour^9,34–43^. Furthermore, chrysomelid beetles possess sexually dimorphic CW^35,38,42–47^, and it has been shown that males use CW as a sex-discriminating cue in several chrysomelid species^35,36,38,39^. However, the mechanisms underlying this sexual discrimination are not fully understood. Especially, there is a lack of knowledge regarding the sensory basis of sexual discrimination in male beetles. Since male sexual discrimination is achieved by the sexually dimorphic CW, it is assumed that female and male CW would elicit different peripheral sensory responses in males, and that such distinct chemosensory inputs would allow males to distinguish between sexes. However, this has not yet been investigated in detail. To better understand the sexual discrimination mechanism, it is necessary to identify the chemosensory organs that males use to detect CW, and investigate how sensory input in response to female and male CW affects male mating decisions.

Our study aimed to elucidate the sensory basis of sexual discrimination in the male strawberry leaf beetle *Galerucella grisescens* (Coleoptera: Chrysomelidae). We used *G. grisescens* as a model chrysomelid beetle since it can be easily reared, and its adults become reproductively mature without diapause, which allows us to prepare a sufficient number of sexually matured adults for the experiments. Through observations of mating behaviour, bioassays for CW, and chemical analyses, we investigated whether males utilised CW as a sex-discriminating cue. We then explored the sensory basis of sexual discrimination using electron microscopy, electrophysiological experiments, and by blocking male chemosensory organs. Our results demonstrate that *G. grisescens* possess qualitatively sexually dimorphic CW, and males rely on chemosensory input from the mouthparts in response to the CW, to distinguish between sexes. To the best of our knowledge, this is the first report of the detailed functional roles of mouthparts in sexual discrimination in the order Coleoptera.

## Results

### Males discriminate conspecific females from males while mounting

**Figure 1:**
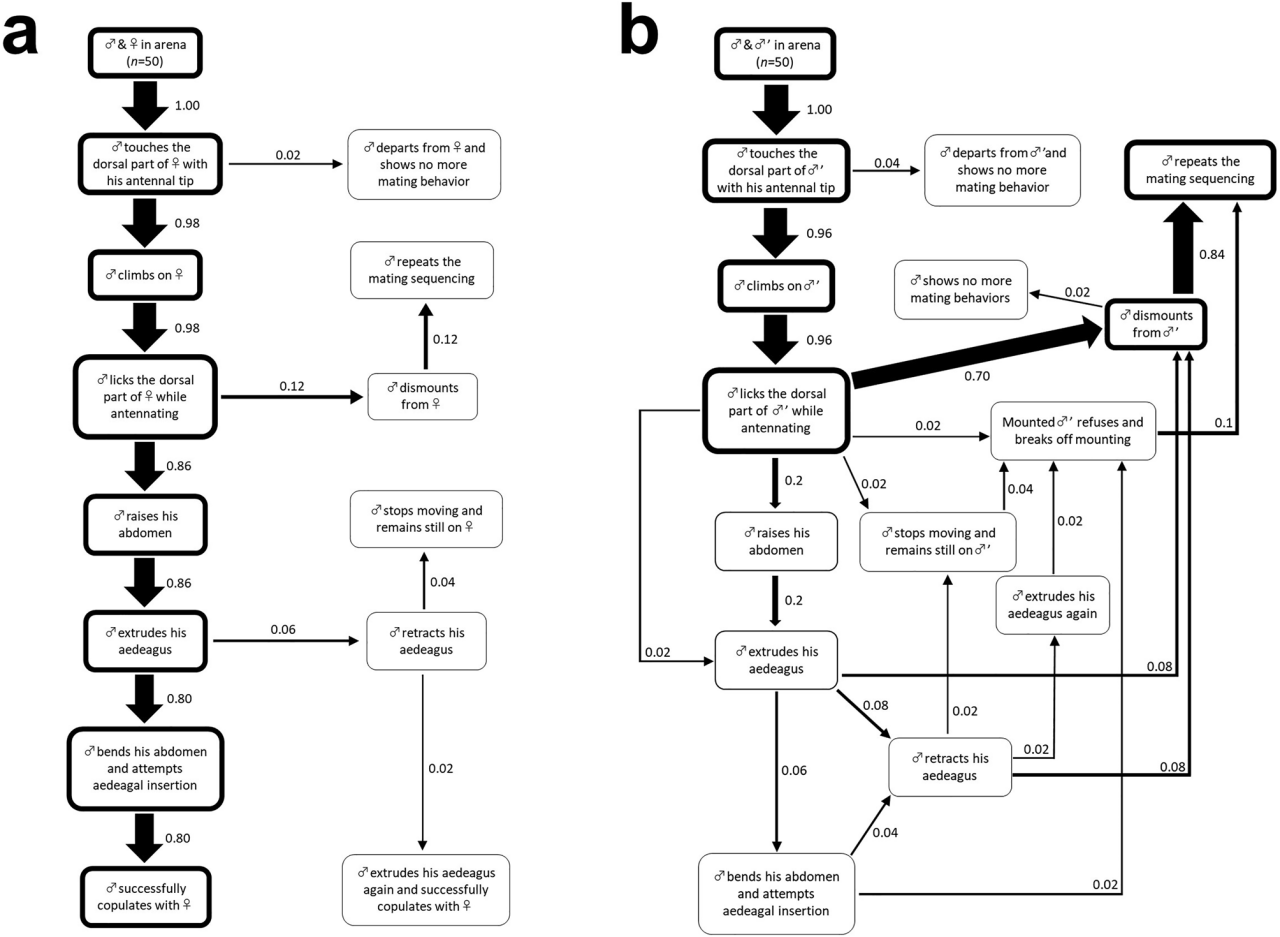
Males discriminate conspecific females from males while mounting. Ethograms of behavioural responses of *Galerucella grisescens* males towards conspecific (**a**) females and (**b**) males (*n* = 50 for each pair). Numerals displayed next to the arrows represent the ratios of pairs that shifted from the behaviour at the start of the arrow to that at the end of the arrow. Thickness of the frames enclosing the behavioural descriptions and the arrows connecting each description are proportional to their ratios.

We first observed male behavioural responses toward conspecific females and males. When males encountered paired females, they performed the following mating sequence: (1) touched the female body with their antennae; (2) mounted and then licked the dorsal part of the female body while antennating; (3) extruded their aedeagus; and (4) attempted to insert their aedeagus into the female genital chamber by bending the abdomen (Fig. 1a). Interestingly, 96% of the males showed mounting behaviour toward paired males in the same manner as they showed towards females (Fig. 1b). However, post-mounting, 90% of the males stopped the mating sequence and dismounted from the males without attempting aedeagal insertion. These results suggest that *G. grisescens* males distinguish between sexes while mounting individuals. Since the dominant behavioural pattern of males after mounting differed between male–female and male-male pairs (i.e., aedeagal insertion toward females and dismounting from males), males probably recognise sex-specific cues in the state of mounting, which help them decide whether to proceed with the mating sequence.

### CW functions as a sex-discriminating cue

**Figure 2:**
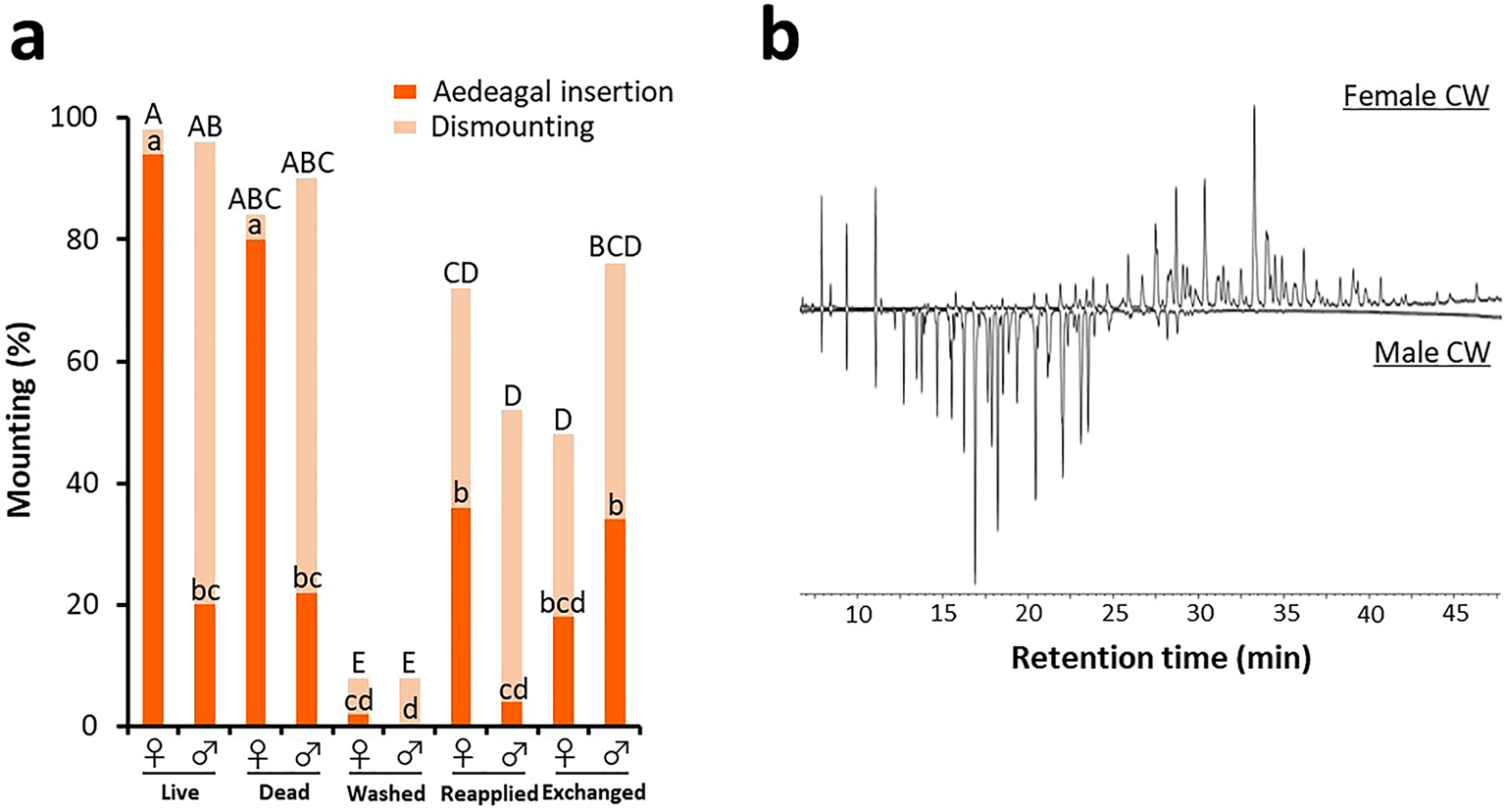
CW functions as a sex-discriminating cue. (**a**) Bioassays for the female and male CW. Bars indicates the percentages of test males that mounted paired potential mates (*n* = 50). The colours on the bars represent male behavioural patterns after mounting: deep and pale red indicate attempting aedeagal insertion and dismounting, respectively. Gender symbols on the x-axis represent the sex of paired potential mates. Treatments to potential mates are shown under gender symbols: live beetles without any treatment (live); freeze-killed (dead); *n*-hexane-washed to remove CW (washed); coated with same- (reapplied) or opposite-sex (exchanged) CW after being washed with *n*-hexane. Same uppercase letters indicate non-significant differences in the percentages of test males that mounted paired potential mates (Fisher’s exact test with Holm correction, *p* > 0.05). Same lowercase letters above the deep red-coloured bars indicate non-significant differences in the percentages of test males that attempted aedeagal insertion toward paired potential mates (Fisher’s exact test with Holm correction, *p* > 0.05). (**b**) Representative total ion chromatograms of the female (above) and male (below) CW, obtained using GC–MS analysis.

To investigate whether the CW functions as a sex-discriminating cue, we first paired males with female or male potential mates with or without CW, and then recorded whether they mounted their paired potential mates. In addition, for pairs in which males showed mounting behaviour, we also recorded the behavioural patterns they showed after mounting—aedeagal insertion or dismounting. Although more than 80% of the males mounted live and dead beetles, regardless of their sex (Fig. 2a), the percentage decreased significantly when the CW of potential mates was removed with *n*-hexane (washed) (Fisher’s exact test with Holm correction, *p* < 0.05). However, once the CW was reapplied, a significantly greater percentage of males mounted than those that had been paired with hexane-washed beetles (Fisher’s exact test with Holm correction, *p* < 0.05), indicating that both female and male CW elicit male mounting behaviour. A significantly greater percentage of males attempted aedeagal insertion toward females than toward males, when both live and dead beetles were presented as potential mates (Fisher’s exact test with Holm correction, *p* < 0.05). A similar trend was observed when the CW of potential mates was reapplied, where a significantly greater percentage of males attempted aedeagal insertion toward females with reapplied CW than toward males with reapplied CW (Fisher’s exact test with Holm correction, *p* < 0.05). However, covering potential mates with heterosexual CW (exchanged) altered male behavioural responses. When hexane-washed males were coated with female CW, the percentage of males attempting aedeagal insertion increased to almost the same level as that observed in case of females with reapplied CW, and was significantly higher than that observed in case of males with reapplied CW (Fisher’s exact test with Holm correction, *p* < 0.05). This suggests that pheromonal activity eliciting male aedeagal insertion is higher in female CW. In contrast, when hexane-washed females were covered with male CW, the percentage of males attempting aedeagal insertion tended to be higher than that observed in case of males with reapplied CW, although the difference was not significant (Fisher’s exact test with Holm correction, *p* > 0.05). Therefore, although CW is essential for sexual discrimination, males may use additional cues. These results suggest that CW functions as a sex-discriminating cue that contributes to the male behavioural decision while mounting—whether to proceed with the mating sequence to aedeagal insertion or dismount.

Because the CW contributes to sexual discrimination, we assumed that the CW profiles in *G. grisescens* would be sex-specific. To confirm our hypothesis, we analysed female and male CW using gas chromatography-mass spectrometry (GC–MS) and observed clear qualitative sexual dimorphism (Fig. 2b). The sexual dimorphism of the CW detected in this study is consistent with its role as a sex-discriminating cue. For males, the CW may be a useful signal to discriminate between sexes, because of its highly sex-specific composition.

### Morphological observation of chemosensory organs using electron microscopy

We observed the male chemosensory organs using electron microscopy, as a preliminary step in identifying CW-detecting organs at the sensillum level. We observed the antennae, tarsi, and mouthparts of adult *G. grisescens* males using scanning (SEM) and transmission electron microscopy (TEM), to examine what types of chemosensory sensilla exist. The terminology and nomenclature used for the classification of the sensilla were based on those used in previous studies^48–51^. Our morphological observations suggest that males possess at least eleven different types of chemosensory sensilla. Their morphological features and distributions are summarised in Table 1.

**Table 1:** Morphological characteristics and distribution of chemosensory sensilla of *Galerucella grisescens* males.

Types^a^	Morphological characteristics					Distribution^c^				
	Length (µm)^b^	Porosity	Surface	Base	Shape	A	T	Mp	Lp	G
Sch I	52.01 ± 2.02	Uniporous	Longitudinal grooves	Socket	Curved	+	+	+	−	−
Sch II	16.68 ± 0.42	Uniporous	Smooth	Socket	Straight	−	−	−	−	+
Sch III	22.16 ± 2.15	Uniporous	Smooth	Socket	Curved	−	−	+	+	−
Sb I	17.01 ± 0.66	Multiporous	Smooth	Convex	Curved	+	−	−	−	−
Sb II	11.11 ± 0.38	Multiporous	Smooth	Convex	Curved/straight	+	−	−	−	−
Sb III	11.89 ± 0.51	Multiporous	Smooth	Convex	Flattened and curved/straight	+	−	−	−	−
Str	27.47 ± 0.49	Multiporous	Longitudinal grooves at basal region Gradually smooth toward distal end	Socket	S-shaped	+	−	−	−	−
Sco	7.77 ± 0.56	Unsure	Veins at upper half region	Convex	Straight	+	−	−	−	−
Sst I	2.41 ± 0.17	Unsure	Veins at distal end	Convex	Straight	−	−	+	+	−
Sst II	1.87 ± 0.04	Uniporous	Smooth	Socket	Straight	−	−	+	+	−
Sca	–	Uniporous	–	–	Concave	+	−	+	+	+

^a^Sch I, Sensilla chaetica subtype I; Sch II, Sensilla chaetica subtype II; Sch III, Sensilla chaetica subtype III; Sb I, Sensilla basiconica subtype I; Sb II, Sensilla basiconica subtype II; Sb III, Sensilla basiconica subtype III; Str, Sensilla trichodea; Sco, Sensilla coeloconica; Sst I, Sensilla styloconica subtype I; Sst II, Sensilla styloconica subtype II; Sca, Sensilla cavity.

^b^Mean values ± SE were obtained from 5–10 individual sensilla of the same type.

^c^A, antennae; T, tarsi; Mp, maxillary palpi; Lp, labial palpi; G, galea; +, present; −, absent.

**Figure 3:**
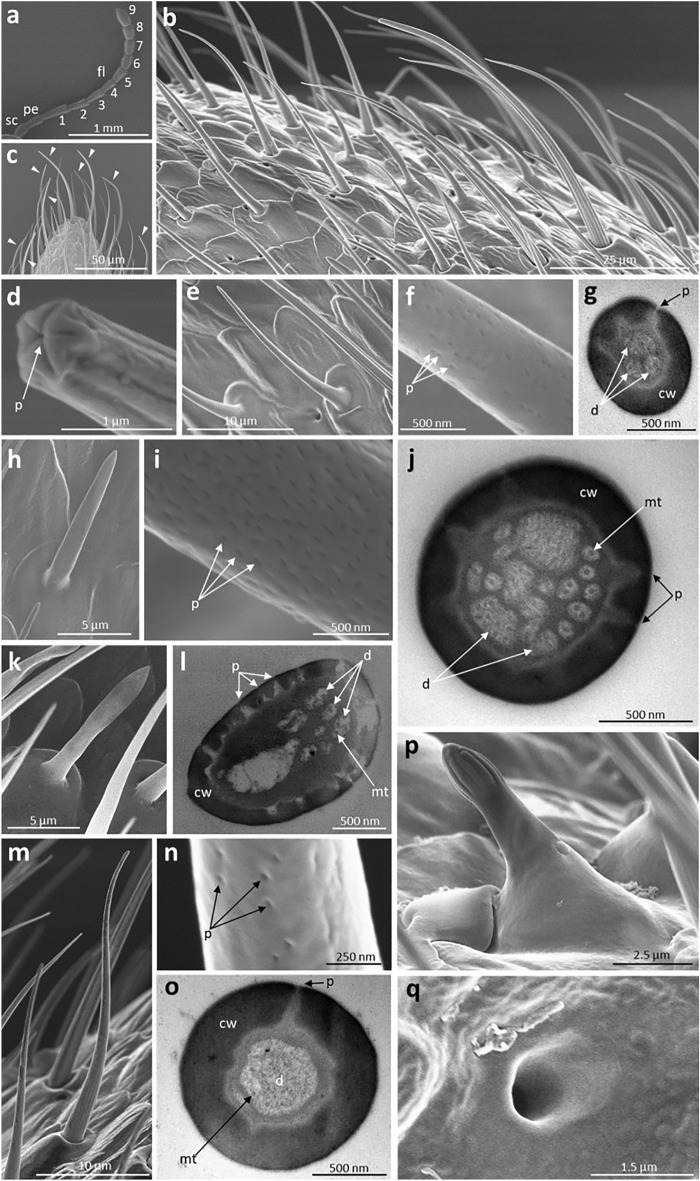
Electron microscopy of male antennae. Outer and inner morphologies were observed using scanning and transmission electron microscope, respectively. (**a**) Whole view of male antennae. sc: scape; pe: pedicel; fl: flagellum; 1–9: the first to ninth flagellomeres. (**b**) Surface of male antennae. (**c**) Distribution of S. chaetica subtype I (arrowheads) on the antennal tip and (**d**) their terminal pores. (**e**) Whole view, (**f**) surface, and (**g**) cross-section of S. basiconica subtype I. (**h**) Whole view, (**i**) surface, and (**j**) cross-section of S. basiconica subtype II. (**k**) Whole view and (**l**) cross-section of S. basiconica subtype III. (**m**) Whole view, (**n**) surface, and (**o**) cross-section at upper region of S. trichodea. (**p**) Whole view of S. coeloconica. (**q**) Whole view of S. cavity. cw: cuticular wall; d: dendrites; mt: microtubules; p: pores.

The antenna consisted of a scape, pedicel, and nine-segmented flagellum bearing numerous sensilla on its surface (Fig. 3a,b). Seven different types of chemosensory sensilla were found, including Sensilla chaetica subtype I, three subtypes of S. basiconica, one subtype of S. trichodea, S. coeloconica, and S. cavity each (Fig. 3c–q). S. chaetica were hair- or thorn-like sensilla inserted into a socket with a terminal pore. Among the three different subtypes of S. chaetica, the antennae bore only S. chaetica subtype I, which were long hair-shaped and set in a round concave socket with a longitudinal grooved wall (Fig. 3c,d, Supplementary Fig. S1). S. basiconica arose from a convex cuticle with a multiporous smooth wall. S. basiconica can be classified into three subtypes: S. basiconica subtype I were slender in shape, with relatively sparse pores on the surface (Fig. 3e–g); Both S. basiconica subtype II and III possessed high-density pores, but they were different in shape. Subtype II were cone-shaped, whereas subtype III had a flattened shaft (Fig. 3h–l, Supplementary Fig. S1). S. trichodea were S-shaped sensilla set in a round, concave socket. Their surfaces in the upper region were multiporous and smooth, while they were longitudinally grooved without any pores in the basal region (Fig. 3m–o, Supplementary Fig. S1). S. coeloconica protruded from the convex cuticle. The surfaces of these sensilla were smooth in the bottom half and deep longitudinally grooved in the top half (Fig. 3p). The S. cavity had round concave pores on the cuticle, without any cuticular projections (Fig. 3q).

Tarsi bore only the S. chaetica subtype I. As previously reported^52^, they were innervated by five sensory neurons (Supplementary Fig. S2).

**Figure 4:**
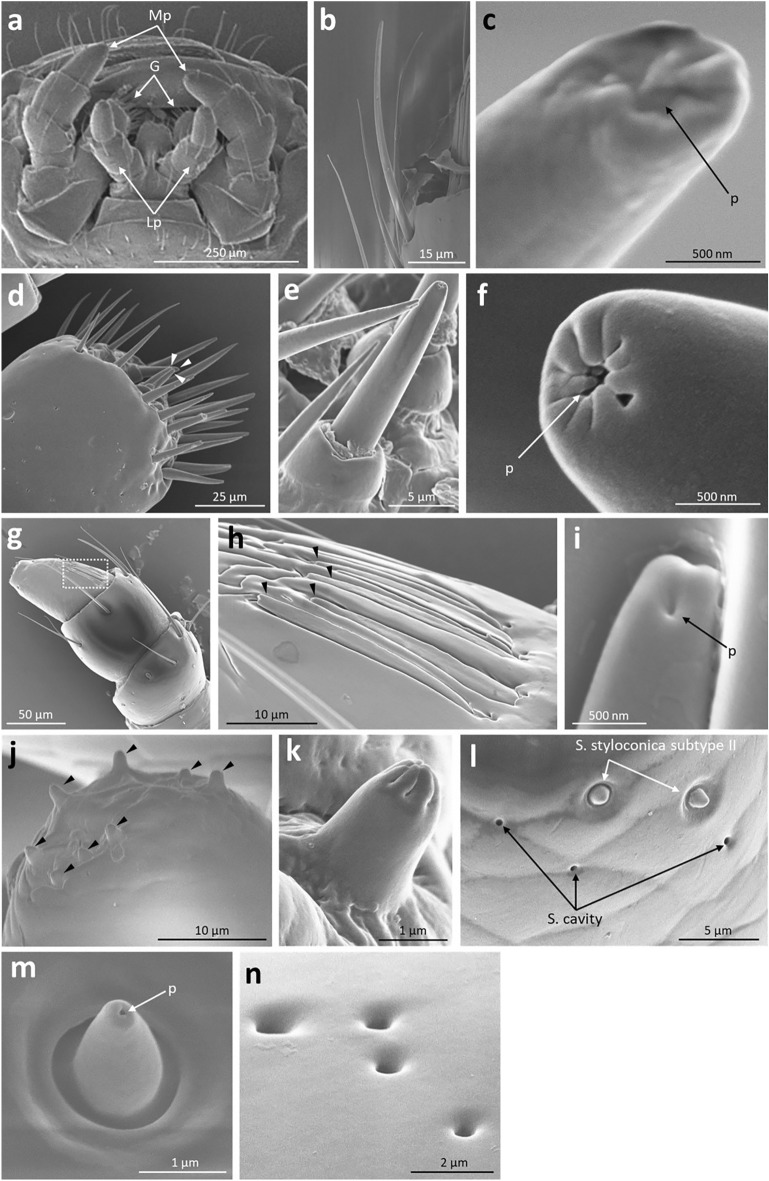
Electron microscopy of male mouthparts. Observations were conducted using scanning electron microscope. (**a**) Gross morphology of male mouthparts. (**b**) Whole view and (**c**) terminal pore of S. chaetica subtype I. (**d**) Distribution of S. chaetica subtype II (arrowheads) on the distal margin of the galea. (**e**) Whole view and (**f**) terminal pore of S. chaetica subtype II. (**g**) Distribution of S. chaetica subtype III on the maxillary palpi (dashed box). (**h**) Higher magnification of the dashed box in (g) showing whole view of S. chaetica subtype III (arrowheads). (**i**) Terminal pore of S. chaetica subtype III. (**j**) Distribution of S. styloconica subtype I (arrowheads) on the tip of the maxillary palpi. (**k**) Whole view of S. styloconica subtype I. (**l**) Surface of the lateral side of the labial palpi. (**m**) Whole view of S. styloconica subtype II. (**n**) Whole view of S. cavity. G: galea; Lp: labial palpi; Mp: maxillary palpi; p: pores.

On the mouthparts, chemosensory sensilla were found on the maxillary palpi, labial palpi, and galea (Fig. 4a). Six sensilla types were observed: three subtypes of S. chaetica, two subtypes of S. styloconica, and one subtype of S. cavity. S. chaetica subtype I were found on the maxillary palpi (Fig. 4b,c). Both S. chaetica subtype II and III were shorter in length than S. chaetica subtype I. S. chaetica subtype II were thorn-shaped sensilla set in a round concave socket with a smoothed wall, and were distributed on the distal margin of the galea (Fig. 4d–f). S. chaetica subtype III were hair-shaped sensilla inserted into a tight socket with a smoothed wall, and were housed in the grooves of the cuticle on the lateral side of the last segment of the maxillary and labial palpi (Fig. 4g–i, Supplementary Fig. S3). S. styloconica were short, peg-shaped, and divided into two subtypes. S. styloconica subtype I possessed deep longitudinal grooves at the distal end, without sockets, and were distributed on the tips of the maxillary and labial palpi (Fig. 4j,k, Supplementary Fig. S3). S. styloconica subtype II were inserted into a round concave socket with a smooth wall and a terminal pore distributed on the lateral side of the maxillary and labial palpi (Fig. 4l,m, Supplementary Fig. S3). S. cavity were sparsely scattered on the maxillary palpi, labial palpi, and galea (Fig. 4l,n, Supplementary Fig. S3).

### Sensory input from the mouthparts while mounting elicits male aedeagal insertion

**Figure 5:**
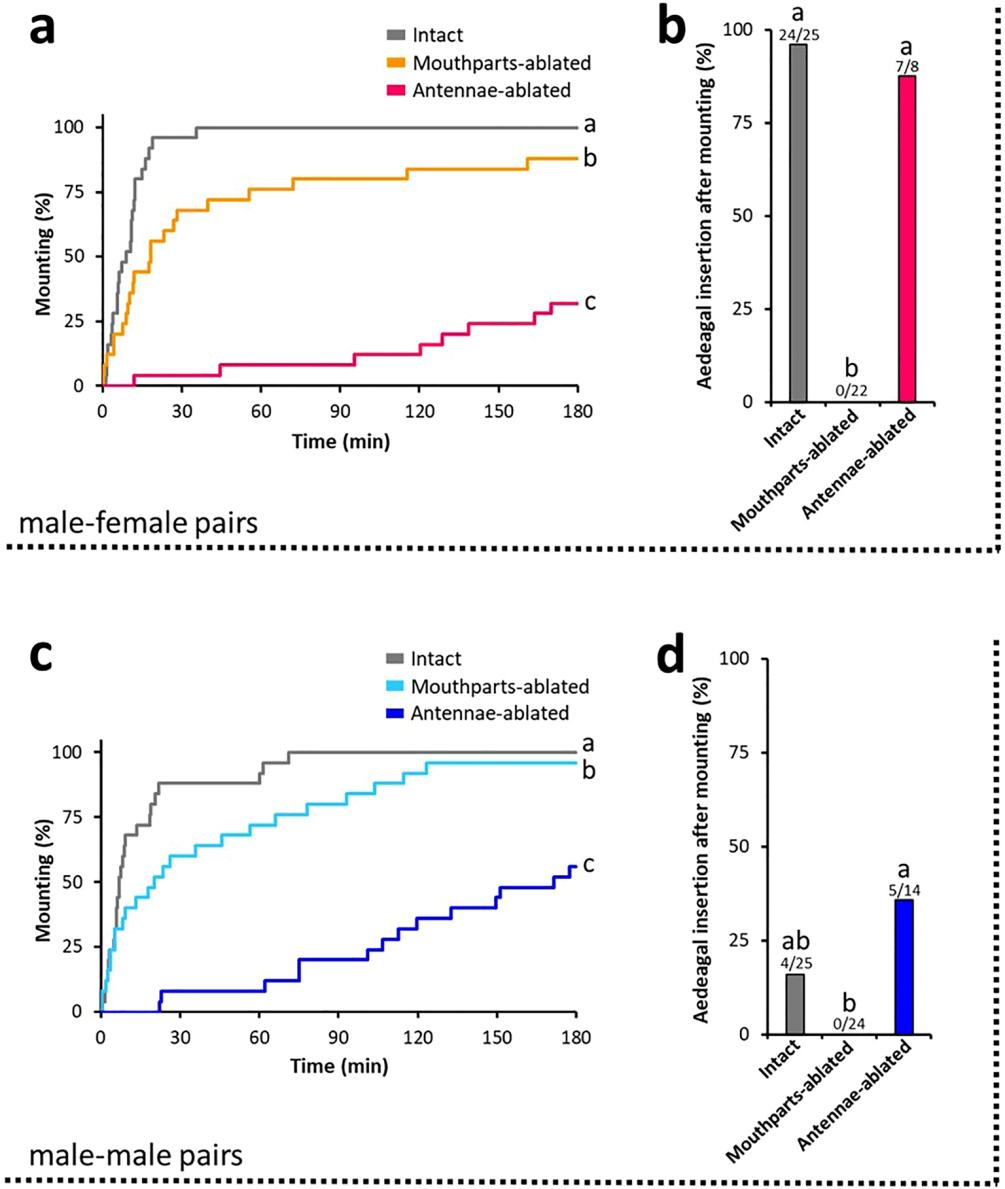
Sensory input from the mouthparts while mounting elicits male aedeagal insertion. Behavioural responses to paired conspecific (**a**-**b**) females and (**c**-**d**) males by males whose antennae or mouthparts bearing chemosensory sensilla (maxillary palpi, labial palpi, and galea) had been ablated (*n* = 25). (**a**) Latency to mount paired females. Same letters indicate non-significant differences (log-rank test with Holm correction, *p* > 0.05). (**b**) Percentages of mounting males that subsequently attempted aedeagal insertion toward paired females. Fractions on the bars represent the number of males that attempted aedeagal insertion out of the number of males that mounted paired females. Same letters indicate non-significant differences (Fisher’s exact test with Holm correction, *p* > 0.05). (**c**) Latency to mount paired males. Same letters indicate non-significant differences (log-rank test with Holm correction, *p* > 0.05). (**d**) Percentages of mounting males that subsequently attempted aedeagal insertion toward paired males. Fractions on the bars represent the number of males that attempted aedeagal insertion out of the number of males that mounted paired males. Same letters indicate non-significant differences (Fisher’s exact test with Holm correction, *p* > 0.05).

Males initiated mating behaviours after touching the cuticle of other individuals with their antennae, following which they licked and antennated while mounting (Fig. 1). These behaviours led us to hypothesise that males receive CW through their antennae and mouthparts. To test this hypothesis, we observed the mating behaviour of *G. grisescens* males in which sensory input from the antennae or mouthparts was eliminated. Either a pair of antennae or mouthparts bearing chemosensory sensilla (maxillary palpi, labial palpi, and galea) were surgically ablated from the males, and the ablated males were then paired with intact females or males as potential mates. We measured the time taken by the ablated males to mount their potential mates. For the pairs in which males mounted within the 180-min timeframe of the experiments, we also recorded whether they subsequently performed aedeagal insertion. In male–female pairs, both antennae- and mouthpart-ablated males showed a significant prolongation of time until mounting, as compared with that observed in case of intact males (log-rank test with Holm correction, *p* < 0.05; Fig. 5a). In particular, antennae-ablated males substantially delayed the initiation of mounting behaviour. Most intact males also shifted to aedeagal insertion (Fig. 5b). In addition, although only 28% of the antennae-ablated males mounted paired females within 180 min, most of them successfully shifted to aedeagal insertion, similar to that observed in case of intact males. However, none of the mouthparts-ablated males performed aedeagal insertion, and the percentage of mounted males that attempted subsequent aedeagal insertion was significantly lower, as compared with that observed in case of the intact and antennae-ablated males (Fisher’s exact test with Holm correction, *p* < 0.05). Similar results were obtained for the male-male pairs (Fig. 5c,d). Both antennae- and mouthparts-ablated males showed a significant prolongation in time until mounting (log-rank test with Holm correction, *p* < 0.05; Fig. 5c), which was greatly extended when the antennae had been ablated. While most intact males dismounted from paired males, 16% mistakenly attempted aedeagal insertion after mounting (Fig. 5d). As with the case of male–female pairs, however, no mouthparts-ablated males performed aedeagal insertion. These results suggest that the antennae and mouthparts play functionally different roles in male mating behaviour. Antennal input is crucial to output mounting behaviour, whereas sensory input from the mouthparts during mounting elicits aedeagal insertion. As the CW regulates these male mating behaviours (Fig. 2a), males probably detect the CW via their antennae and mouthparts. Because sensory input from the mouthparts elicits male aedeagal insertion, a common male behavioural response to females, male mouthparts are likely to be tuned to aphrodisiac components in the female CW.

### Male mouthparts bear chemosensory sensilla tuned to female CW components

**Figure 6:**
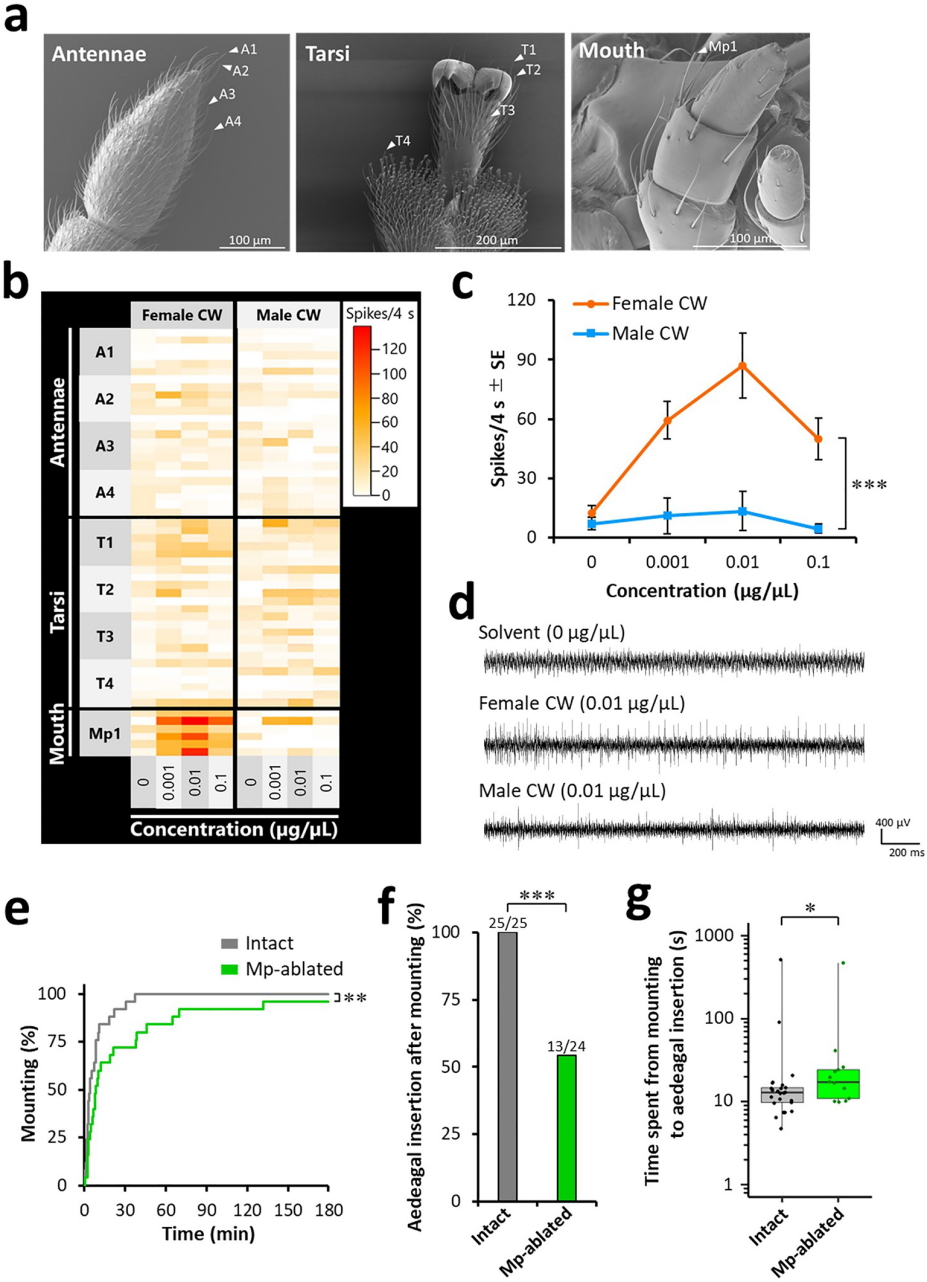
Male mouthparts bear chemosensory sensilla tuned to female CW components. (**a**) Scanning electron micrographs of the targeted sensilla (arrowheads) in the electrophysiological experiments. S. chaetica subtype I on the male antennae, tarsi, and mouthparts were targeted. (**b**) Neural responses of the targeted sensilla to the female and male CW. The targeted sensilla were stimulated with a series of ascending concentrations of the female or male CW solutions. Each recording was continued for 4 s. The number of spikes recorded in the 4 s was counted. Each of the four cells continuously arranged in the horizontal direction in the heatmap indicates each replicant. The right and left halves of the heatmap represent neural responses to the male and female CW, respectively. A total of 108 males were used (9 targeted sensilla × 2 CW types × 6 replicates). (**c**) Neural responses of Mp1 to the female and male CW. Asterisks indicate a significant difference between the types of CW (GLMM Type III ANOVA, ***p < 0.001). (**d**) Representative recorded neural responses of Mp1 to the control solvent (above), female CW (middle), and male CW (below). (**e**–**g**) Behavioural responses to paired conspecific females by males whose sensory input from S. chaetica subtype I on the mouthparts had been eliminated by ablating their maxillary palpi (Mp) (*n* = 25). (**e**) Latency to mount paired females. A significant difference has been indicated using asterisks (log-rank test, **p < 0.01). (**f**) Percentages of mounting males that subsequently attempted aedeagal insertion toward paired females. Fractions on the bars represent the number of males that attempted aedeagal insertion out of the number of males that mounted paired females. A significant difference has been indicated using asterisks (Fisher’s exact test, ***p < 0.001). (**g**) Time spent from initiating mounting behaviour to attempting aedeagal insertion by males that attempted aedeagal insertion toward paired females. Significant differences have been indicated using asterisks (Mann–Whitney *U* test, *p < 0.05).

We hypothesised that male mouthparts would possess chemosensory sensilla that are tuned to female CW components and conducted electrophysiological experiments to test the same. Among the six different types of chemosensory sensilla found on male mouthparts (Table 1), S. chaetica subtype I were chosen as the target sensilla because their morphological characteristics resemble those of the gustatory sensilla of *Drosophila melanogaster*, whose function is to detect pheromonal components in the CW^53–55^. Therefore, we recorded the neural responses of S. chaetica subtype I on the male mouthparts to CW (Fig. 6a). This type of sensilla was also distributed on the antennae and tarsi (Table 1), and their responses were recorded for comparison (Fig. 6a). Although the antennal and tarsal sensilla did not evoke clear responses to CW, those on the mouthparts (Mp1) did evoke activated neural responses to female CW in all replicates (Fig. 6b–d). Mp1 responded significantly higher to female CW than to male CW [generalised linear mixed model (GLMM) Type III analysis of variance (ANOVA), χ^2^ = 16.5, *df* = 1, *p* < 0.001]. The interaction between CW type and concentration also had a significant effect on neural responses to Mp1 (GLMM Type III ANOVA, χ^2^ = 14.0, *df* = 1, *p* < 0.001), although concentration did not (GLMM Type III ANOVA, χ^2^ = 0.295, *df* = 1, *p* = 0.587). These results suggested that the S. chaetica subtype I on the mouthparts are CW-detecting organs that are particularly tuned to female CW components.

Next, we confirmed the findings of our electrophysiological experiments through behavioural observations. In the male mouthparts, S. chaetica subtype I were distributed only on the maxillary palpi (Table 1). Thus, we eliminated the sensory input by ablating a pair of maxillary palpi and observed the behavioural responses of the ablated males toward paired intact females. Ablated males showed significantly delayed mounting (log-rank test, *p* < 0.01, Fig. 6e), and importantly, a significant reduced percentage of these proceeded to aedeagal insertion after mounting (Fisher’s exact test, *p* < 0.001; Fig. 6f). These results indicate that S. chaetica subtype I on male maxillary palpi detect female CW. Although a significant decrease was observed, approximately 54% of the ablated males that had mounted still performed aedeagal insertion (Fig. 6f). However, the influence of eliminating sensory input was observed even in ablated males that shifted to aedeagal insertion. There was a significant increase in time from initiating mounting behaviour to attempting aedeagal insertion (Mann–Whitney *U* test, *p* < 0.05; Fig. 6g), which implied that the male behavioural decision was disturbed by eliminating female pheromonal input from S. chaetica subtype I on the maxillary palpi. These results support our finding that males detect female CW components via S. chaetica subtype I on the mouthparts.

## Discussion

Previous studies have suggested that the CW functions as a sex-discriminating cue in Chrysomelidae^35,36,38,39^. However, the sensory basis for sexual discrimination in male beetles remains unclear. Our study revealed that chemosensory input from the mouthparts mediated sexual discrimination in *G. grisescens* males.

When males encounter conspecific individuals, they inspect them by licking their body surfaces. While in case of females, the males proceed to aedeagal insertion, in case of males, they dismount (Fig. 1). We found that such male behavioural decisions are regulated by the qualitatively sexually dimorphic CW (Fig. 2). We also revealed that males attempted aedeagal insertion when they received sensory input from the mouthparts while mounting (Fig. 5). Consistent with this, electrophysiological experiments carried out in this study showed that chemosensory sensilla on male mouthparts, namely S. chaetica subtype I, detect female CW components (Fig. 6). These results suggest that males receive pheromonal input from the mouthparts while licking the cuticle of females, which must be an essential aphrodisiac signal for proceeding the male mating sequence to aedeagal insertion. On the other hand, males seem to receive only weak input while mounting males, which may lead them to end their mating attempts toward males. The licking behaviour of males during mating sequences has been reported across diverse families of the order Coleoptera: Chrysomelidae^9,40,56^, Cerambycidae^12,57^, Curculionidae^58^, Coccinellidae^11^, Lampyridae^59^, Silvanidae^60^, Buprestidae^61^, Anobiidae^62^, Dermestidae^63,64^, and Scarabaeidae^10^. However, the mating cues that they detect via their mouthparts have not yet been clarified. Our study provides direct evidence that male mouthparts can be used to detect the CW components in females. To our knowledge, this is the first detailed report of the functional roles of mouthparts in male mate recognition in Coleoptera. Although there is a need for further studies, males of other Coleopteran species may also use their mouthparts to detect CW components, as numerous studies have demonstrated that male beetles rely on the female CW for mate recognition^9–19^.

We found that S. chaetica subtype I on the male mouthparts detect female CW components. However, the results of our study imply that males may also use other chemosensory sensilla to receive CW. Although males without maxillary palpi, labial palpi, and galea could not proceed to aedeagal insertion (Fig. 5b, d), approximately half of the males in which only the maxillary palpi had been ablated did (Fig. 6f). These results indicate that males use additional sensilla on their mouthparts to detect the CW, because S. chaetica subtype I were distributed only on the maxillary palpi at their mouthparts. Moreover, our results imply that males may first roughly recognise potential mates by detecting CW components via their antennae, and then initiate mounting behaviour if they accept them. Consistent with this assumption, a previous study reported that the antennae of *Zygogramma bicolorata* (Coleoptera: Chrysomelidae) males evoke neural responses to shared CW components between females and males^44^. Because activated neural responses were not recorded in the antennal S. chaetica subtype I in our electrophysiological experiments, males may have used other types of sensilla. To the best of our knowledge, the types of sensilla used by coleopteran insects to detect CW are yet to be reported. This information is available for other insect orders, such as ants^65^, hornets^66^, and fruit flies^67^, which have been reported to receive CW via antennal basiconic sensilla. Electron microscopy revealed that *G. grisescens* males possessed the same type of sensilla (Fig. 3e–l, Supplementary Fig. S1). Thus, they may use the antennal S. basiconica to detect CW components. There is a need for further investigations to fully identify the chemosensory sensilla that receive CW.

In Chrysomelidae, it has been reported that female and male CW profiles are qualitatively very similar or the same, and the sexual dimorphism of CW is quantitative rather than qualitative; that is, females and males share the same set of CW components, but in different relative quantities^35,38,42–46^. Although the identification of pheromonal components in the CW has not been vigorously advanced in Chrysomelidae, it is suggested that males rely on sexual differences in the abundance ratios of shared CW components between females and males for sexual discrimination^38^. This assumption is consistent with the fact that insect pheromones often consist of several compounds and are functional only when each compound is mixed in a certain proportion^1^. In contrast, our GC–MS analysis revealed that most CW components of *G. grisescens* are sex-specific. To the best of our knowledge, our study is the first to report significant qualitative sexual dimorphism of CW in Chrysomelidae. Since sex-specific components are likely to be useful for sexual discrimination, the presence or absence of sex-specific CW components, rather than sexual differences in the abundance ratios of shared CW components, may be important for *G. grisescens* males to discriminate between sexes. In fact, when CW is qualitatively sexually dimorphic, males use female-specific components as aphrodisiac pheromones in several species of Cerambycidae^68–70^, a family closely related to Chrysomelidae. To elucidate the components of CW that *G. grisescens* males use, we are currently carrying out qualitative and quantitative analyses of their CW and identification of pheromonal components.

Although our bioassays showed that *G. grisescens* males used CW for mate recognition, reapplying CW did not perfectly restore their behavioural responses towards live and dead beetles (Fig. 2a). Previous studies have reported a similar partial reduction in behavioural responses to reapplied CW^12,13,24,38,57^. Although the obvious causes have not yet been elucidated, the original spatial distribution pattern of CW components^71,72^ and nanoscale topology of CW^73,74^ are likely to be disrupted upon reapplication, which may reduce male behavioural responses towards the beetles with reapplied CW. Another possible explanation is that whole-body extracts might contain not only CW components but also inner components from the body of test insects, and such potential contaminants negatively affected male behavioural responses. Furthermore, when hexane-washed beetles were coated with heterosexual CW, the male behavioural responses were not completely reversed, as compared with those observed in the beetles with reapplied CW. Specifically, when hexane-washed females were covered with male CW, the percentage of males that attempted aedeagal insertion tended to be higher than that observed in male beetles with reapplied CW, although the difference was not significant (Fig. 2a). One possible reason for this is that males make use of additional polar aphrodisiac compounds on the bodies of females, which are difficult to remove with *n*-hexane. Consistent with our assumption, it has been suggested that males of some coleopteran species use synergistic polar compounds as well as non-polar CW components such as long-chain hydrocarbons^43,75^. Future studies should focus on identifying additional aphrodisiac cues in females.

In conclusion, our work demonstrates that chemosensory input from the mouthparts in response to the qualitatively sexually dimorphic CW mediates sexual discrimination in *G. grisescens* males. To the best of our knowledge, this is the first report describing the sensory basis of CW-driven sexual discrimination and detailed functional roles of mouthparts in mate recognition in Coleoptera. We anticipate that our study will provide important insights into mate recognition systems in Coleoptera and encourage further studies on insect chemical communication.

## Methods

### Insects

*Galerucella grisescens* were maintained in a climate chamber at 25 ± 1 °C and 60% relative humidity, under a 16L:8D photoperiod. They were reared on fresh leaves of *Rumex obtusifolius*. To obtain unmated adult beetles, newly emerged adults were sexed and maintained separately. Unmated adults at 7–8 days post-emergence were used in the experiments.

### Extraction of CW

CW was extracted from 100 females and 100 males per extraction. Beetles were freeze-killed at − 20 °C, following which they were thawed for 15 min at room temperature. Afterwards, they were immersed in two sequential 20 mL aliquots of *n*-hexane, for 30 min each. The two aliquots were filtered through filter paper and mixed. The solvent was evaporated at 39 °C using a rotary vacuum evaporator, and the resulting extract was dried under a gentle stream of nitrogen gas. The CW obtained was used for bioassays, chemical analyses, and electrophysiological experiments.

### Male behavioural responses toward conspecific females and males

Individual males were paired with either individual females or males in a 48-mm-diameter glass Petri dish lined with filter paper. The behavioural responses of the test males were recorded for 3 h at 25 ± 1 °C during the light period of the rearing condition. While observing male-male pairs, only focal males (observation targets) were marked with black paint on their elytra, to differentiate them from non-focal partner males. Ethograms for each female-male and male-male pair were constructed based on male behavioural responses in the first mating attempt (*n* = 50). The style of the ethograms was partly referred to in previous studies^76,77^.

### Bioassays for CW

A male was released on a 48-mm-diameter glass Petri dish lined with filter paper, on which a potential mate was fixed with double-sided tape. Potential mates were either conspecific females or males subjected to one of the following four treatments: 1) dead, 2) washed, 3) reapplied, or 4) exchanged. For treatment 1, live beetles were killed at –20 °C for 30 min, following which they were thawed for 15 min at room temperature. For treatment 2, the CW of freeze-killed beetles was removed with *n*-hexane using the same procedure described above for CW extraction. The solvent was then allowed to evaporate for 30 min. For treatment 3, CW was reapplied by pipetting one beetle equivalent of the *n*-hexane solution of CW that had been extracted from beetles of the same sex onto hexane-washed beetles. After pipetting CW solution, the solvent was allowed to evaporate for 30 min. For treatment 4, one beetle equivalent of the opposite-sex CW solution was pipetted onto hexane-washed beetles. The solvent was then allowed to evaporate for 30 min. Male behavioural responses to paired potential mates were recorded at 25 ± 1 °C during the light period of the rearing condition. We observed whether the males mounted their potential mates (*n* = 50). Furthermore, for the pairs in which males mounted, we also recorded the behavioural pattern they subsequently showed after mounting: aedeagal insertion or dismounting. The bioassays were terminated when males dismounted from potential mates three times in a row or attempted aedeagal insertion, which were regarded as ‘dismounting’ and ‘aedeagal insertion’, respectively. If males did not exhibit mounting behaviour within 3 h, they were regarded as having no response. In addition to the four treatments of potential mates mentioned above, male behavioural responses to live beetles were added to the results for comparison (as a control).

### Chemical analyses

The CW samples were reconstituted in 500 µL of *n*-hexane. Five samples were prepared from each sex. An aliquot of 1 µL of each sample was injected into a GC–MS (GCMS-QP2010 Ultra, Shimadzu, Kyoto, Japan) equipped with a DB-5 ms column (30 m × 0.25 mm i.d., 0.25 µm film thickness, J&W Scientific, CA, USA). The analytical conditions have been described in a previous study^9^. Helium was used as a carrier gas at a column head pressure of 100 kPa. The inlet temperature was set to 280 °C and injection was performed in split mode (1:20). The oven temperature programme was started at 50 °C and heated to 230 °C, at a rate of 30 °C/min, then increased to 320 °C at a rate of 2 °C/min, and finally held at 320 °C for 30 min. The mass spectrometer was operated in electron impact mode, at 70 eV.

### SEM

After male beetles were freeze-killed at − 20 °C for 30 min, the bodies were thawed for 15 min at room temperature and then washed in distilled water using an ultrasonic cleaner. The antennae, heads, and tarsi were dissected from their bodies and mounted on cylindrical stubs with double-sided carbon tape, under a light microscope. The samples were left in a desiccator at least overnight, followed by coating with platinum-palladium using an ion sputter coater MSP-1S (Vacuum Device, Ibaraki, Japan). The specimens were examined using a scanning electron microscope SU8000 Type II (Hitachi, Tokyo, Japan). The length of each sensilla type was obtained from 5–10 individual sensilla. Measurements were conducted using ImageJ software version 1.53t (National Institutes of Health, Bethesda, MD, USA).

### TEM

The following procedure has been used in a previous study^52^: Briefly, the male beetles were washed with distilled water using an ultrasonic cleaner. The antennae and tarsi were then removed and fixed in 2.5% glutaraldehyde solution in 0.2 M phosphate buffer (PB) pH 7.0, overnight, at 4 °C. After primary fixation, the samples were rinsed thrice in PB and immersed in 1% osmium tetroxide for 2 h post-fixation. Subsequently, the samples were rinsed once in PB, dehydrated in a graded ethanol series (50%–100%), and embedded in Quetol 812-Araldite resin (Nisshin EM, Tokyo, Japan) using propylene oxide as a bridging solvent. Ultrathin Sects. (80 nm) were cut with a diamond knife on an ultramicrotome UltraCut S (Leica Microsystems GmbH, Wetzlar, Germany), collected on formvar-coated copper grids, and double-stained with TI Blue and lead citrate. Sample grids were examined using a transmission electron microscope (H-7650 ZeroA; Hitachi).

### Blocking male chemosensory organs

A pair of antennae or mouthparts bearing chemosensory sensilla (maxillary palpi, labial palpi, and galea) were surgically removed from the males using forceps, 1 h prior to the experiments. The ablated males were paired with either intact conspecific females or males in a 48-mm-diameter glass Petri dish lined with filter paper (*n* = 25). We then measured the time taken by the ablated males to mount their paired potential mates. For pairs in which ablated males mounted, we also recorded whether they subsequently attempted aedeagal insertion. The observations continued for a maximum of 3 h. The assays were conducted at 25 ± 1 °C during the light period of the rearing condition. Following this procedure, we conducted experiments using males whose only maxillary palpi had been ablated (*n* = 25). In this case, the ablated males were paired with only females, and the time spent from initiating mounting behaviour to attempting aedeagal insertion was measured, in addition to the two investigation items mentioned above.

### Electrophysiological experiments

A tip-recording method^78^ was used to record the neural responses of S. chaetica subtype I to female and male CW. Nine sensilla were targeted: four sensilla on the antennae (A1, A2, A3, and A4), four on the foretarsi (T1, T2, T3, and T4), and one on the maxillary palpus (Mp1). Recordings were conducted during the light period under the rearing conditions. The experimental setup was based on a previous study^52^. The test males were CO_2_-anesthetised, and their midlegs, hindlegs, and wings were removed from their bodies using forceps. A pair of mandibles, pair of labial palpi, and left maxillary palpus were additionally removed when Mp1 was targeted. The males were then fixed onto a glass slide with a kneaded eraser. Glass electrodes were pulled from borosilicate glass capillaries (1.5 mm o.d., 1.1 mm i.d., 10 cm length, with filament) using a micropipette puller P-97 (both from Sutter Instrument, CA, USA). A glass capillary filled with 100 mM KCl was inserted into the ventral side of the thorax between the prosternum and mesosternum, which acted as the reference electrode. The recording electrode was filled with a stimulus solution of female or male CW that had been dissolved in the control solvent (5 mM KCl with 0.1% Triton™ X-100) using an ultrasonic cleaner^65^. The targeted sensillum was stimulated with a series of ascending concentrations of the stimulus solutions, by covering its tip with a recording electrode using a micromanipulator MHW-103 (Narishige Scientific Instrument Lab., Tokyo, Japan), under observation using a stereomicroscope (SteREO Discovery V20; Carl Zeiss, Oberkochen, Germany). Recordings were started simultaneously with stimulus onset and continued for 4 s. To avoid potential adaptation of chemosensory neurones, the tip of the sensilla was washed with distilled water during an interstimulus interval of 3 min. The recorded signals were amplified using a Taste Probe DTP-02, filtered with IDAC-4, and analysed using AutoSpike software v3.7 (all from Ockenfels Syntech GmbH, Buchenbach, Germany). Neural responses were evaluated by counting the number of spikes generated during the 4-s recording (*n* = 6). Each test male was recorded once, and a total of 108 males were used (nine targeted sensilla × two CW types × six replicants).

### Statistical analysis

The percentages of males that showed mounting behaviour and those that attempted aedeagal insertion in the bioassays were compared using Fisher’s exact test with Holm correction. In the experiments where the male chemosensory organ was blocked, the latency to mount paired conspecific individuals was compared using the log-rank test, with Holm correction used for multiple comparisons. The percentages of mounting males that shifted to aedeagal insertion were compared using Fisher’s exact test, and Holm correction was used for multiple comparisons. The time from initiating mounting behaviour to attempting aedeagal insertion was compared using the Mann–Whitney *U* test. Type III ANOVA using the GLMM procedure was used to test the effect of CW type, concentration, and their interaction on the neural responses obtained from Mp1 in the electrophysiological experiments. The types of CW, concentrations, and interactions between them were fixed effects, and each test male was included as a random effect. The model assumed a Poisson distribution and used a log-link function. All statistical analyses were performed using R version 4.1.2^79^.

### Supplementary Information

Supplementary Figures.

## Data Availability

The datasets are available from the corresponding author on reasonable request.
