# Supplementary material for: Chemosensory input from mouthparts in response to sexually dimorphic cuticular wax mediates male sexual discrimination in Galerucella grisescens (Coleoptera: Chrysomelidae)
Source: Sci Rep. 2023 Dec 8;13:21754. doi: 10.1038/s41598-023-49272-1 (PMC10709455; doi:10.1038/s41598-023-49272-1)
Supplement: Supplementary file 1 — Supplementary Figures. [file 41598_2023_49272_MOESM1_ESM.pdf]

Chemosensory input from mouthparts in response to sexually dimorphic cuticular wax mediates male sexual discrimination in *Galerucella grisescens* (Coleoptera: Chrysomelidae)

Yuki Chiba[1], Shun Yosano[2], Masatoshi Hori[1*]

[1]Graduate School of Agricultural Science, Tohoku University, Sendai, Miyagi 980-8572, Japan

[2]Institute for Plant Protection, National Agriculture and Food Research Organization, Tsukuba, Ibaraki 305-8666, Japan

[*]Corresponding author: masatoshi.hori.a3@tohoku.ac.jp

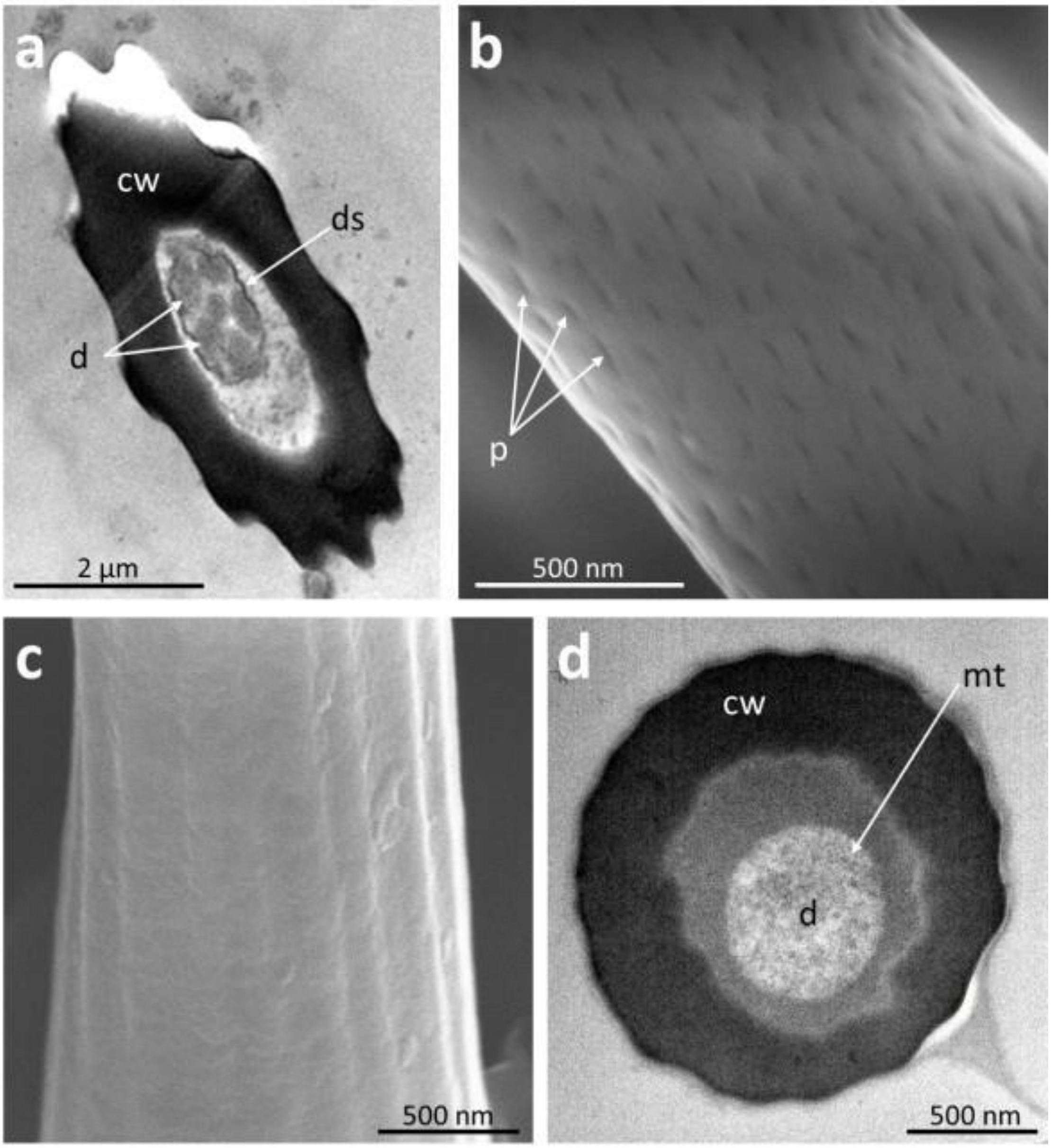

Supplementary Figure S1. Electron microscopy of male antennae. Outer and inner morphologies were observed using scanning and transmission electron microscope, respectively. (**a**) Cross section of S. chaetica subtype I. (**b**) Surface of S. basiconica subtype III. (**c**) Surface and (**d**) cross section of S. trichodea close to the base. cw: cuticular wall; d: dendrites; ds: dendritic sheath; mt: microtubules; p: pores.

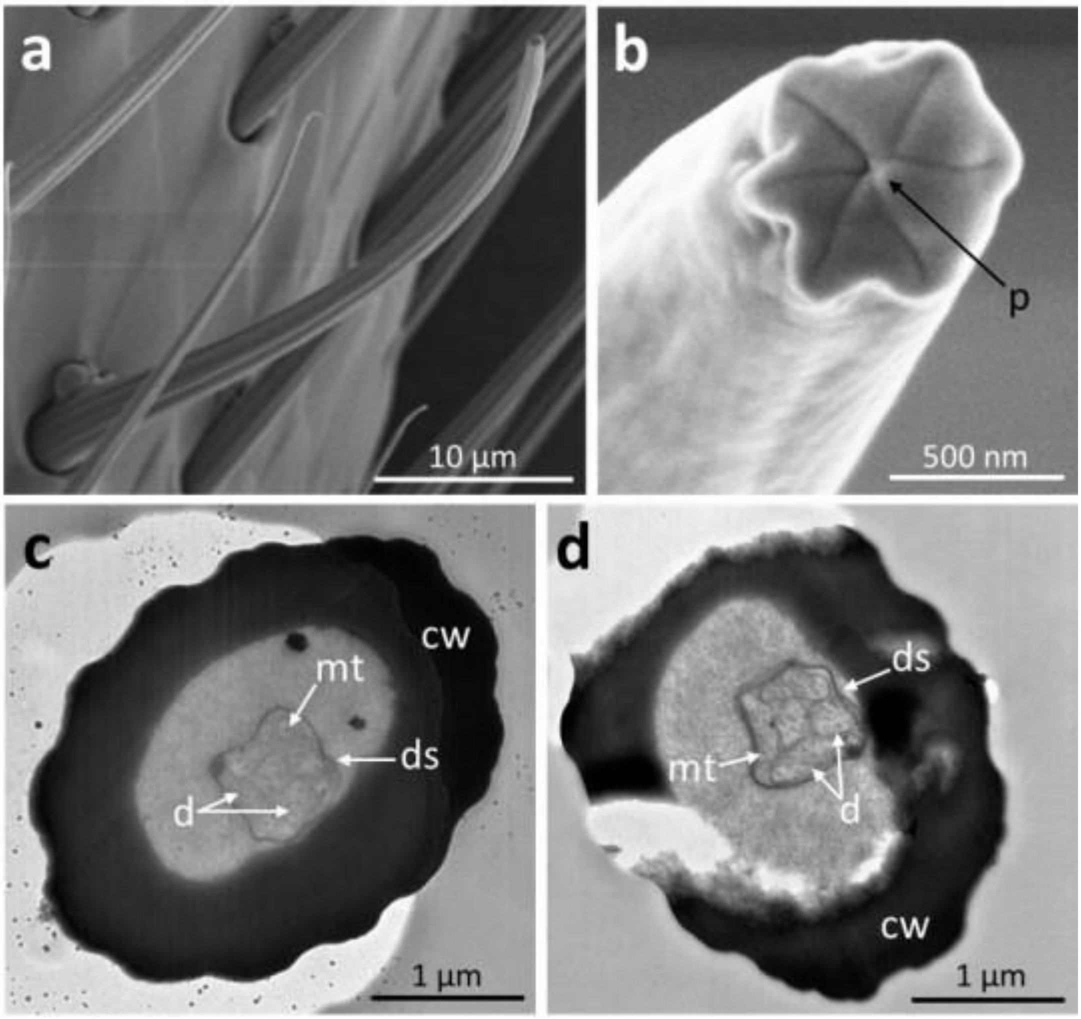

Supplementary Figure S2. Electron microscopy of male tarsi. Outer and inner morphologies were observed using scanning and transmission electron microscope, respectively. (**a**) Whole view, (**b**) terminal pore, and (**c**, **d**) cross section of S. chaetica subtype I. cw: cuticular wall; d: dendrites; ds: dendritic sheath; mt: microtubules; p: pores.

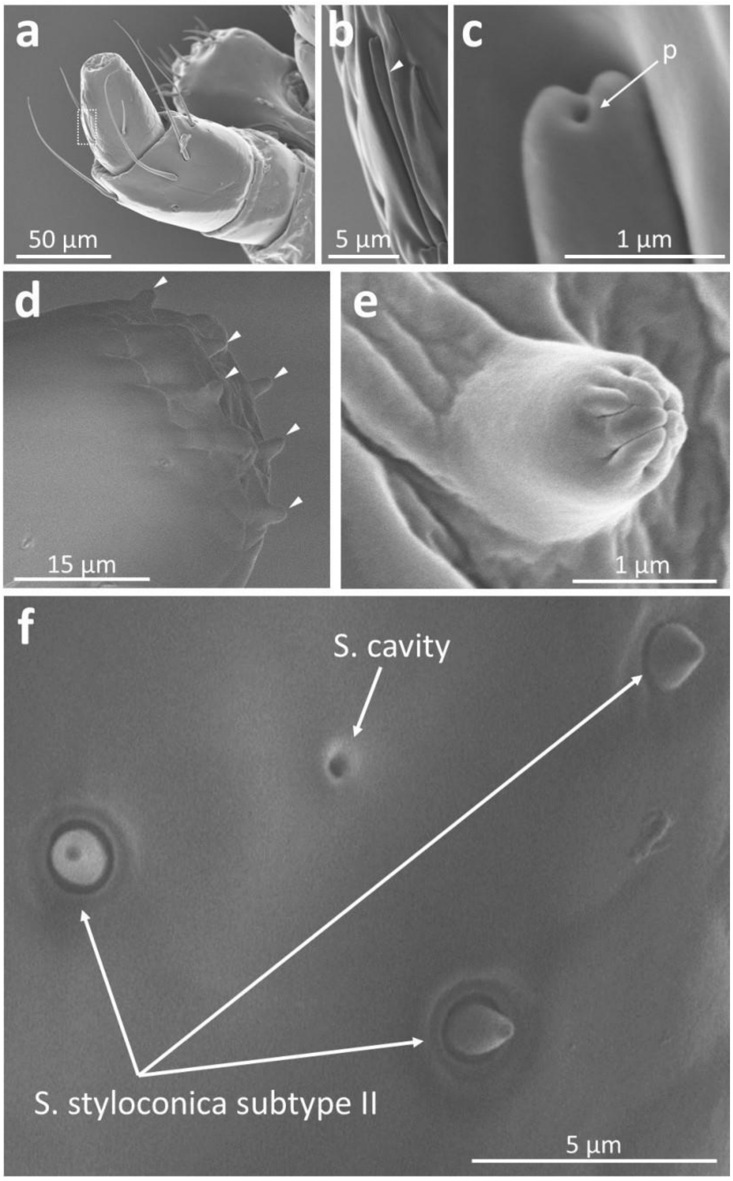

Supplementary Figure S3. Electron microscopy of male mouthparts. Observations were conducted using scanning electron microscope. (**a**) Distribution of S. chaetica subtype III on the labial palpi (dashed box). (**b**) Higher magnification of the dashed box in (a) showing whole view of S. chaetica subtype III (arrowhead). (**c**) Terminal pore of S. chaetica subtype III. (**d**) Distribution of S. styloconica subtype I (arrowheads) on the tip of the labial palpi. (**e**) Top view of S. styloconica subtype I. (**f**) Surface of the lateral side of the maxillary palpi. p: pores.
